# The “SpiDa” dataset: self-report questionnaires and ratings of spider images from spider-fearful individuals

**DOI:** 10.3389/fpsyg.2024.1327367

**Published:** 2024-05-30

**Authors:** Alexander Karner, Mengfan Zhang, Cindy Sumaly Lor, David Steyrl, Sebastian Jakob Götzendorfer, Steffi Weidt, Filip Melinscak, Frank Scharnowski

**Affiliations:** ^1^Department of Cognition, Emotion, and Methods in Psychology, University of Vienna, Vienna, Austria; ^2^Department of Psychiatry, Psychotherapy and Psychosomatics, University of Zurich, Zurich, Switzerland

**Keywords:** spider phobia, arachnophobia, fear of spiders, anxiety, fear, disgust, approach-avoidance

## 1 Introduction

Spider phobia is characterized by intense and irrational aversive reactions to spiders (American Psychiatric Association, 2013). With a prevalence between 2.7 and 9.5% (e.g., Fredrikson et al., 1996; Oosterink et al., 2009; Zsido, 2017) it is one of the most common types of specific phobias (Wardenaar et al., 2017), particularly in females (Fredrikson et al., 1996). Considering the widespread presence of spiders in both natural and urban environments (e.g., Bolger et al., 2000; Magura et al., 2010; Argañaraz and Gleiser, 2017), spider phobia can have a highly negative impact on one's quality of life (e.g., Choy et al., 2007). Two emotions that are strongly associated with spider phobia are fear and disgust (Olatunji et al., 2011). From an evolutionary perspective, while fear is thought to serve the purpose of preventing potential harm (Griffith, 1919; Fanselow, 1984; World Health Organization, 2018), disgust has been linked to the prevention of contamination or the contraction of infectious diseases (Matchett and Davey, 1991; Olatunji and Sawchuk, 2005; Olatunji and McKay, 2006). It is well-documented that the perception of negative emotional stimuli leads to avoidance responses (e.g., Solarz, 1960), with exaggerated avoidance being a characteristic symptom of specific phobias (American Psychiatric Association, 2013). Unsurprisingly, spider-fearful individuals have been shown to exhibit increased avoidance behavior toward spiders and are hesitant to approach them (Rinck and Becker, 2007). However, individuals who want to overcome their fear may be willing to approach a feared stimulus, resulting in an approach-avoidance conflict (Lewin, 1935).

Research tools have been developed to investigate the characteristics of spider phobia, including a variety of self-report questionnaires designed to measure subjective levels of fear of spiders (e.g., Watts and Sharrock, 1984; Szymanski and O'Donohue, 1995), as well as disgust (e.g., Schienle et al., 2002, 2010). Additionally, fear, disgust, and approach-avoidance responses can be induced in humans with the help of visual stimuli (e.g., Matthews et al., 2010; Haberkamp et al., 2017; Rinck et al., 2021), with spider images evoking particularly strong responses for fear and disgust (Gerdes et al., 2009). Being able to characterize the subjective levels of these psychological dimensions in spider-fearful individuals is key to treatments that aim to reduce aversive responses through repeated exposure (Craske et al., 2014; Benito and Walther, 2015). To present exposure stimuli in a systematic way, it is useful to have knowledge of the aversive response that stimuli elicit. This knowledge also provides a foundation for computerized, exposure-based treatments (e.g., Matthews et al., 2010). Such treatments have the potential to become an easily accessible treatment option in the future.

In this data report, we present the “SpiDa” dataset, which consists of data from an online survey, in which spider-fearful individuals filled out a variety of self-report questionnaires indicating their level of fear of spiders (Rinck et al., 2002), disgust propensity (Schienle et al., 2002), disgust sensitivity (Schienle et al., 2010), and constructs such as state- and trait anxiety (Spielberger et al., 1970; Laux et al., 1981). Participants then rated spider images according to “fear,” “disgust,” and “willingness to approach” and underwent a follow-up after 1 week. Here, we describe the methods that were used to obtain the dataset and provide the dataset alongside an extensive codebook with descriptive statistics.

## 2 Materials and methods

### 2.1 Overview of experimental design

The online survey was composed of a main survey and a short follow-up. The main survey started with the completion of several self-report questionnaires (see Section 2.3). At this point participants filled out the German adaptation of the Fear of Spiders Questionnaire (FSQ; Szymanski and O'Donohue, 1995; Rinck et al., 2002) for the first time (FSQ_1). Subsequently, participants underwent an image rating phase consisting of 100 rating trials in which they rated images depicting spider-related content according to relevant psychological dimensions. After the first half of rating trials, as well as after the second half, participants filled out a break questionnaire stating their current levels of fear, exhaustion, boredom, physical arousal, and disgust. Participants then rated five neutral images before they answered the FSQ a second time (FSQ_2). One week after the main survey, participants were invited to a short follow-up, in which they filled out the FSQ a third time (FSQ_3). A graphical overview of the experimental design is provided in the Supplementary Figure 1.

### 2.2 Participants

Participants who took part in this online survey were 203 healthy German-speaking individuals (157 female, 45 male, one diverse) between the ages of 18 and 45 (mean = 23.36, SD = 4.40), with self-reported fear of spiders and willingness to overcome their fear (i.e., “Are you afraid of spiders? Are you ready to overcome your fear?”). Out of these participants, 192 (148 female, 43 male, one diverse; mean age = 23.39, SD = 4.43) completed the main survey. Participants were recruited from a university participant database. Each participant signed up to take part via the university's online system for study participation (Sona Systems, https://www.sona-systems.com) and received a link to the online survey on the University of Vienna's “SoSci Survey” (Leiner, 2020) platform. As the study was conducted online, participants had the flexibility to choose both a convenient time and location to complete the survey. They were asked to concentrate on the study and avoid any distractions such as music or mobile phones. Before the start of the experiment, participants were informed that physical and mental agitation was to be expected due to the presentation of fear-inducing stimuli, and that they could withdraw from the study at any point. Moreover, participants were instructed to stop filling out the survey in case of troublesome symptoms. Exclusion criteria were pregnancy, past or present diagnosed psychiatric illnesses, or a history of alcohol or drug abuse. The study was conducted in German. Participants either received a financial compensation (*n* = 152; 7 € for the main survey, 2 € for the follow-up), or course credits (*n* = 40). The median time needed to complete the main survey was 38 min. The median time needed to complete the follow-up was 3 min. Informed consent was obtained before the start of the experiment. Data were securely collected and stored without the involvement of any third parties. Participants agreed to their data being made available in online repositories in anonymized form. The data were collected between December 2020 and May 2021.

### 2.3 Self-report questionnaires

Participants stated their gender, age, and current country of residence, and subsequently answered psychometric self-report questionnaires, which were presented to them in the following order: (1) the validated German version of the Fear of Spiders Questionnaire (Szymanski and O'Donohue, 1995; Rinck et al., 2002), (2) the German adaptation of the Spider Phobia Questionnaire (SPQ; Watts and Sharrock, 1984; Rinck et al., 2002), (3) the German “Spinnenangst-Screening” questionnaire (SAS; Rinck et al., 2002), (4) the German adaptation of State Trait Anxiety Inventory (STAI; Spielberger et al., 1970; Laux et al., 1981), (5) a disgust propensity questionnaire (FEE; Schienle et al., 2002), and (6) a questionnaire indicating disgust sensitivity (SEE; Schienle et al., 2010). The order stated above was maintained throughout the study. However, the items of each questionnaire were presented in randomized order to 98 participants (group 1) while 94 participants were presented with the questionnaires' standard item order (group 2), allowing the investigation of item order effects (Sahin, 2021). An overview of the self-report questionnaires is provided in Supplementary Table 1, including sum score ranges and reliability measures. Sum scores are provided in the dataset.

### 2.4 Stimuli

After filling out self-report questionnaires, participants rated stimuli from a study by Zhang et al. (2024) according to three psychological dimensions. The stimuli consisted of 313 images showing spiders, or spider-related content. These images were selected with the aim to induce different levels of fear in spider-fearful individuals, which resulted in a heterogeneous image database, covering a broad variety of spider-related content, such as cobwebs, cartoon spiders, small spiders, large spiders, spiders eating prey, or spiders that are in physical contact with humans. Each image had a size of 800 x 600 pixels and was assigned with an individual image ID.

### 2.5 Image rating procedure

For each participant, 95 images were randomly sampled without replacement from a list that included 372 images (313 unique images and 59 duplicates). The duplicate images were included to enable assessing the effects of repeated exposures. In each image rating trial, an image was presented on the screen for 3 s. Subsequently the image disappeared, and 3 questions and corresponding rating scales were presented on the screen. (1) “How much fear does this picture elicit in you?” (2) “How much disgust does this picture elicit in you?” Questions 1 and 2 were the same for all participants. However, question 3 varied between the groups. For participants in group 1, question 3 was formulated as “How close could you come to the content shown in the picture, if you wanted to overcome your fear?” whereas the question was phrased as “How close could you come to the content shown in the picture if you wanted to overcome your aversion?” in group 2. Ratings were administered by moving a continuously adjustable slider on a 101-point visual analog scale, one of which was positioned below each question. For questions 1 and 2, ratings were administered on a scale from 0 to 100% in increments of 1%. Question 3 was rated on a scale from 0 to 10 m in increments of 0.1 m (with 0 m labeled as “touch,” and 10 m labeled as “distance”). Participants were given 12 s to administer all three ratings. Once they had administered the three ratings, they could click “Next” in the bottom-right corner on the screen to proceed with the next image. If they failed to answer the questions within 12 s, questions that had not been answered yet were marked in red and the following warning message appeared on the screen: “Please also answer this question—your answer to this question is very important for the study.” Participants could only proceed with the next trial after administering all three ratings. The order of the three questions remained constant throughout the survey and was identical for all participants. Moreover, the survey included catch trials, in which participants were instructed to position the slider to either the left or the right end of the scale for each of the three rating scales (e.g., “Please move the slider to the left end of the fear scale, to the left end of the disgust scale, to the right end of the distance scale”) in different combinations. A catch trial was accepted as correct when the ratings for all three scales were administered according to the instructions within a tolerance region of 5% at each end of the “fear” and “disgust” scale, and “0.5 m” at each end of the “approach” scale, respectively. Screenshots of the image rating procedure and the catch trials are provided.

### 2.6 Image rating phase

After being presented with instructions on the rating procedure, participants underwent some practice rounds, which consisted of three trials with neutral images that showed inanimate objects (such as a fork, shoes, a stone, a sponge, or a pencil on neutral background), followed by one catch trial. Then, they were presented with the instructions on the rating procedure a second time and asked to click “Start” once they were ready to start rating spider-related images.

The image rating phase consisted of 100 trials, composed of 95 rating trials with randomly chosen images from the image database as described above, as well as two neutral images and three catch trials in-between. The positions of neutral rating trials and catch trials were the same for all participants. After 50 rating trials, as well as after all 100 rating trials, participants answered a break questionnaire, in which they were asked to state their current level of fear, exhaustion, boredom, physical arousal, and disgust. For each of the items, participants had to administer a rating on a subjective units of distress scale (Wolpe, 1969) from 0 to 10, with 0 being the minimal and 10 being the maximal manifestation of each attribute. Like the psychometric questionnaires, the break questionnaire was administered in randomized item order in group 1, and in a fixed item order in group 2. Each break questionnaire contained a bogus item (e.g., Meade and Craig, 2012). After completing the image rating phase and the second break questionnaire, participants underwent five additional rating trials with neutral images.

### 2.7 Post-rating-, and follow-up questionnaire

After the image rating phase, participants filled out the FSQ a second time. The completion of the post-rating questionnaire marked the end of the main survey. Seven days after the main survey, participants received a link to a follow-up, in which they were given a time window of 24 h to fill out the FSQ a third time.

### 2.8 Data preparation

The dataset contains responses from all participants who finished at least the main survey, regardless of whether they also finished the follow-up. Participants who only partially responded to the main survey were excluded. Four participants only completed the main survey the second time and ended it after a small number of questions on the first attempt. The data of their first attempt was excluded. Additionally, the second follow-up response of one participant who completed the follow-up questionnaire twice was excluded. To anonymize and prepare the data for sharing, ID codes and the variable “current country of residence” were removed. The data were cleaned and processed in R (R Core Team, 2023) using RStudio (Posit team, 2023). Moreover, the dataset includes the time in seconds that each participant needed to administer image ratings, complete the self-report questionnaires and the entire survey, respectively. The corresponding codebook was generated with the “codebook” package (Arslan, 2019) and includes distribution plots, summary statistics and value labels for each variable. In addition to the original SpiDa dataset, we also provide a filtered dataset that includes data of participants who had an FSQ score ≥ 24 (Rinck and Becker, 2007), answered all bogus items correctly and did not fail more than one catch trial during the image rating phase, resulting in a total of *n* = 152 participants (see data repository for details). Moreover, we include a dataset containing the trial-wise image rating data of these participants.

## 3 Dataset overview

**Figure 1:**
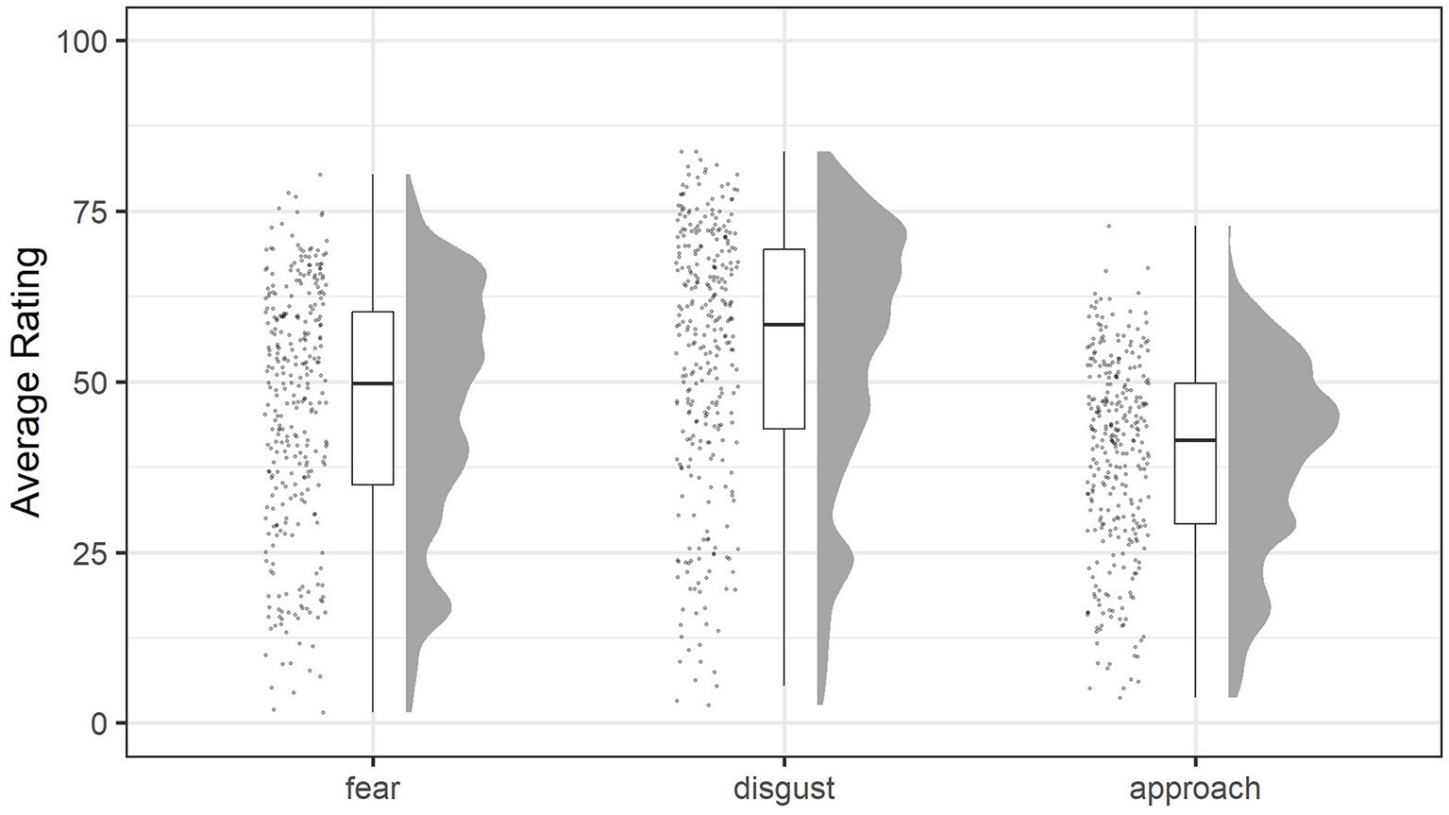
Average fear-, disgust-, and approach-ratings per spider image across all participants, calculated from the rating data obtained during the image rating phase. Images were rated on 101-point scales. Specifically, the scales of fear and disgust had a range from 0 to 100% (rated in increments of 1%), while the approach scale had a range from 0 to 10 m (rated in increments of 0.1 m).

A total 192 participants completed the main survey, 153 of which additionally completed the follow-up. Table 1 presents the descriptive statistics of questionnaire sum scores that were calculated based on individual question items. Summary statistics of questionnaire sum scores of group 1 and group 2 are provided in Supplementary Table 2. The main survey mean questionnaire sum scores of group 1 and group 2 were very highly correlated (*r*_s_ = 0.976, *n* = 192, *p* < 0.001). Figure 1 shows the distribution of average fear-, disgust- and approach-ratings per spider image across all participants, calculated from the rating data obtained during the image rating phase. Approach-ratings of group 1 and group 2 were pooled to compute the average ratings.

**Table 1:** Summary statistics of questionnaire sum scores.

**Variable**	***n***	**Mean**	**Median**	**Std. dev**.	**Min**	**Max**
FSQ_1_sum	192	50.37	54	23.57	1	108
FSQ_2_sum	192	59.09	60	27.50	0	108
FSQ_3_sum	153	46.31	46	26.73	0	103
SPQ_sum	192	13.10	13	6.22	0	29
SAS_sum	192	14.88	16	5.59	0	24
STAI_state_sum	192	42.71	40.5	12.02	23	80
STAI_trait_sum	192	44.24	43	10.23	23	71
FEE_sum	192	89.29	88.5	18.22	46	148
SEE_sum	192	16.66	16	5.61	7	34

Mean and standard deviation (std. dev.) were rounded to two decimal places.

## 4 Limitations

The present study has several limitations. Firstly, we did not include additional mental health screening information in addition to the self-report questionnaires. Secondly, the data collection of groups 1 and 2 was performed consecutively, and not in a randomized manner. Thirdly, our stimuli were not standardized regarding colors or objects, which might potentially have affected the image ratings. Finally, although participants were encouraged to focus on the study and avoid any distractions such as music or mobile phones, we cannot rule out that some were distracted or did not properly pay attention to the study, as they completed the survey online.

## 5 Value and use of the data

The data are beneficial for researchers studying fear of spiders and may be used to investigate a broad variety of research questions, and for exploratory analyses. Among others, the dataset enables researchers to further examine associations between psychometric self-report questionnaires and the psychological dimensions of subjective ratings of fear, disgust, and approach-avoidance. FSQ responses, which include follow-up data, can be used to investigate potential habituation effects driven by the repeated exposure to spider images. The ratings of duplicated images can be employed to examine the effects of repeated exposure to the same image. Additionally, the data may be used to investigate item order effects in self-report questionnaires (e.g., Sahin, 2021). The data can be filtered according to questionnaire cutoff scores, number of catch trial fails or false responses to bogus items. Further investigations could apply a similar approach in other populations such as in different countries or with clinically diagnosed individuals.

## Data availability statement

The datasets presented in this study can be found in online repositories. The names of the repository/repositories and accession number(s) can be found at: https://osf.io/zw2yg/.

## Ethics statement

The studies involving humans were approved by the Ethics Committee of the University of Vienna. The studies were conducted in accordance with the local legislation and institutional requirements. The participants provided their written informed consent to participate in this study.

## Author contributions

AK: Writing—original draft, Conceptualization, Data curation, Investigation, Software, Visualization, Formal analysis, Methodology. MZ: Data curation, Writing—review & editing, Validation, Software. CL: Conceptualization, Methodology, Writing—review & editing. DS: Conceptualization, Writing—review & editing, Methodology. SG: Conceptualization, Methodology, Writing—review & editing. SW: Conceptualization, Writing—review & editing. FM: Data curation, Writing—review & editing. FS: Conceptualization, Resources, Supervision, Writing—review & editing, Methodology.
