# Supplementary material for: The “SpiDa” dataset: self-report questionnaires and ratings of spider images from spider-fearful individuals
Source: Front Psychol. 2024 May 30;15:1327367. doi: 10.3389/fpsyg.2024.1327367 (PMC11169805; doi:10.3389/fpsyg.2024.1327367)
Supplement: Supplementary file 1 [file Table_1.pdf]

Supplementary Material

The "SpiDa" dataset: Self-report questionnaires and ratings of spider images from spider-fearful individuals

Alexander Karner[1], Mengfan Zhang[1], Cindy Sumaly Lor[1], David Steyrl[1], Sebastian Jakob Götzendorfer[1], Steffi Weidt[2], Filip Melinscak[1], Frank Scharnowski[1*]

[1]Department of Cognition, Emotion, and Methods in Psychology, University of Vienna, Vienna, Austria

[2]Department of Psychiatry, Psychotherapy and Psychosomatics, University of Zurich, Zurich, Switzerland

*** Correspondence:** Frank Scharnowski: frank.scharnowski@univie.ac.at

Supplementary Figure 1. Overview of the experimental design.

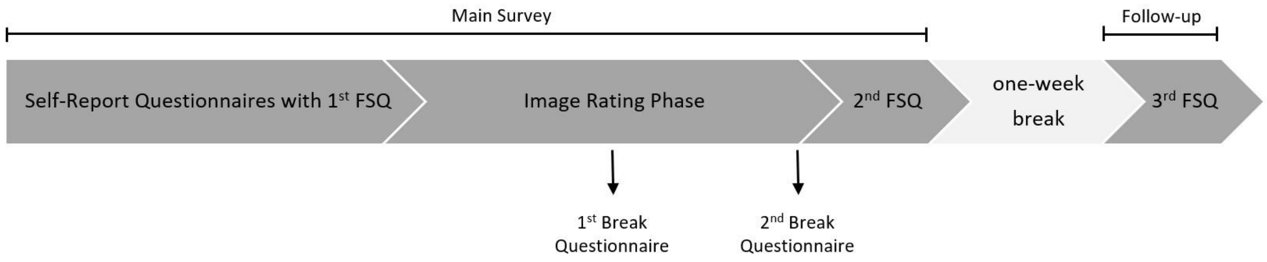

Supplementary Table 1. Overview of self-report questionnaires used in the present study. All questionnaires were previously validated through correlations with related scales (see "Reference"); the FSQ, SPQ and SAS (Rinck et al., 2002) were additionally validated through a behavioral test where participants approached a live spider.

Questionnaire	Rating Scale	Number of Items	Sum score range (present study)	Cronbach's alpha (original publication)	Reference
FSQ	7-point	18	0 - 108	0.96	Rinck et al. (2002)
SPQ	no / yes	43	0 - 33	0.84	Rinck et al. (2002)
SAS	7-point	4	0 - 24	0.92	Rinck et al. (2002)
STAI_state	4-point	20	20 - 80	0.90 (for both STAI scales)	Laux et al. (1981)
STAI_trait	4-point	20	20 - 80		Laux et al. (1981)
FEE	5-point	37	0 - 148	0.90	Schienle et al. (2002)
SEE	5-point	7	7 - 35	0.85	Schienle et al. (2010)

Supplementary Table 2. Summary statistics of questionnaire sum scores of group 1 (gr. 1; 75 female, 23 male; mean age = 23.05, SD = 4.80, min = 18, max = 45) and group 2 (gr. 2; 73 female, 20 male, 1 diverse; mean age = 23.74, SD = 4.01; min = 18, max = 42). Mean and standard deviation (Std. Dev.) were rounded to two decimal places.

	**n**		**Mean**		**Median**		**Std. Dev.**		**Min**		**Max**	
**Variable**	gr. 1	gr. 2	gr. 1	gr. 2	gr. 1	gr. 2	gr. 1	gr. 2	gr. 1	gr. 2	gr. 1	gr. 2
FSQ_1_sum	98	94	50.35	50.39	54	54.5	25.17	21.90	1	4	108	95
FSQ_2_sum	98	94	58.97	59.21	59.5	60.5	28.73	26.32	0	3	108	108
FSQ_3_sum	74	79	47.81	44.90	49	44	26.94	26.62	0	1	101	103
SPQ_sum	98	94	12.97	13.23	12	13	6.50	5.94	0	1	29	29
SAS_sum	98	94	14.70	15.06	15	16	5.95	5.22	2	0	24	24
STAI_state_sum	98	94	43.98	41.39	43	38	11.70	12.25	24	23	80	75
STAI_trait_sum	98	94	43.72	44.78	43	45	9.20	11.23	26	23	65	71
FEE_sum	98	94	88.76	89.85	90	86.5	19.57	16.78	48	46	148	124
SEE_sum	98	94	16.57	16.76	16	16	5.83	5.40	7	7	32	34

References

Laux, L., Glanzmann, P., Schaffner, P., and Spielberger, Charles, D. (1981). *Das State-Trait-Angstinventar.* Göttingen: Beltz.

Rinck, M., Bundschuh, S., Engler, S., Müller, A., Wissmann, J., Ellwart, T., and Becker, E. S. (2002). Reliabilität und Validität dreier Instrumente zur Messung von Angst vor Spinnen. *Diagnostica* 48, 141–149. doi: 10.1026//0012-1924.48.3.141.

Schienle, A., Dietmaier, G., Ille, R., and Leutgeb, V. (2010). Eine Skala zur Erfassung der Ekelsensitivität (SEE). *Zeitschrift für Klinische Psychologie und Psychotherapie* 39, 80–86. doi: 10.1026/1616-3443/a000016.

Schienle, A., Walter, B., Stark, R., and Vaitl, D. (2002). Ein Fragebogen zur Erfassung der Ekelempfindlichkeit (FEE). *Zeitschrift für Klinische Psychologie und Psychotherapie* 31, 110–120. doi: 10.1026/0084-5345.31.2.110.
